# High-Quality Draft Genome Sequence of *Candida apicola* NRRL Y-50540

**DOI:** 10.1128/genomeA.00437-15

**Published:** 2015-06-11

**Authors:** Leticia Vega-Alvarado, Jorge Gómez-Angulo, Zazil Escalante-García, Ricardo Grande, Anne Gschaedler-Mathis, Lorena Amaya-Delgado, Alejandro Sanchez-Flores, Javier Arrizon

**Affiliations:** a Unidad de Biotecnología Industrial, Centro de Investigación y Asistencia en Tecnología y Diseño del Estado de Jalisco A.C., Jalisco, Mexico; b Unidad de Secuenciación Masiva y Bioinformática, Instituto de Biotecnología de la UNAM, Morelos, México

## Abstract

*Candida apicola*, a highly osmotolerant ascomycetes yeast, produces sophorolipids (biosurfactants), membrane fatty acids, and enzymes of biotechnological interest. The genome obtained has a high-quality draft for this species and can be used as a reference to perform further analyses, such as differential gene expression in yeast from *Candida* genera.

## GENOME ANNOUNCEMENT

*Candida apicola*, a highly osmotolerant ascomycetes yeast, produces sophorolipids (biosurfactants), membrane fatty acids, and enzymes, such as reductases and proteases (1–3). Naturally, this yeast has been found in wine and cachaça fermentation processes. In cachaça fermentation, it exhibits the capacity to produce volatile compounds (4–6). Recently, it was found that *C. apicola* yeasts isolated from the mezcal fermentation process secreted β-fructofuranosidases with fructosyltransferase activity, useful for prebiotic synthesis. Therefore, this fermentation process could reveal certain interesting features that can be discovered by performing whole-genome sequencing of *C. apicola*. It could lead us to a specific gene catalogue for biotechnological applications in fermentation bioprocesses.

Genomic DNA from *C. apicola* NRRL Y-50540 (YPD culture) was isolated and prepared as Illumina sequencing libraries to generate a total of 13,207,584 paired-end reads (estimated coverage ~211×) with a length of 72 bases, using the Illumina GAIIx platform. The assembly was performed with Velvet v1.2.10 using a k-mer size of 51. An assembly of 9,769,876 bp in 40 contigs with lengths greater than or equal to 1,000 bp, was obtained with *N*_50_/*N*_90_ values of 773,945/186,965 bp, respectively (7). The average contig length was 107,585 bp, giving a considerable space to search for genes. The average G+C content was 41.6%, which is similar to that of other *Candida* species. Gene prediction was performed using Augustus v2.7 (8) using three different *Candida* species profiles (*C. albicans*, *C. guilliermondii*, and *C. tropicalis*). We predicted 3,818 protein-coding genes by intersecting all three predictions. Using CEGMA v2.5 (9), we obtained 92% genome completeness. A group of enzymes from other yeast species, with functions related to carbohydrate polymer synthesis and degradation, such as secretory aspartyl protease (SAP2p), exoinulinases, and invertases, are present in the genome. However, the best hits for these proteins have only ~35% identity, implying a high divergence at the sequence level and probably several modifications in terms of substrate recognition and activity compared to those present in other yeasts found in fermentation processes.

Therefore, the *C. apicola* genome presented here is, to our knowledge, the first high-quality draft genome for this species and can be used as a reference to perform further analyses, such as differential gene expression of enzymes related to the synthesis and degradation of biotechnological molecules of interest, for example, in different fermentation conditions, which is one of our main interests. The availability of these genomes can also contribute to the understanding of the *Candida* genus, which is arguably biased by the amount of information related to the opportunistic human pathogen *Candida albicans*.

### Nucleotide sequence accession numbers.

This whole-genome shotgun project has been deposited at GenBank under the accession numbers LBNK01000001 to LBNK01000040.
