# Correction for Vega-Alvarado et al., High-Quality Draft Genome Sequence of *Candida apicola* NRRL Y-50540 

**DOI:** 10.1128/genomeA.01043-15

**Published:** 2015-08-27

**Authors:** Leticia Vega-Alvarado, Jorge Gómez-Angulo, Zazil Escalante-García, Ricardo Grande, Anne Gschaedler-Mathis, Lorena Amaya-Delgado, Alejandro Sanchez-Flores, Javier Arrizon

**Affiliations:** a Unidad de Biotecnología Industrial, Centro de Investigación y Asistencia en Tecnología y Diseño del Estado de Jalisco A.C., Jalisco, México; b Unidad de Secuenciación Masiva y Bioinformática, Instituto de Biotecnología de la UNAM, Morelos, México; c Centro de Ciencias Aplicadas y Desarrollo Tecnológico, UNAM, México City, México

## AUTHOR CORRECTION

Volume 3, no. 3, e00437-15, 2015. Page 1: The author affiliations should read as given above.

